# Measles Outbreak Associated with an Infectious Traveler — Colorado, May–June 2025

**DOI:** 10.15585/mmwr.mm7504a1

**Published:** 2026-01-29

**Authors:** Amanda R. Metz, Meghan Barnes, Kevin Andresen, Ginger Stringer, Nicole Comstock, Alexis Burakoff, Shannon R. Matzinger, Leslee Warren, Marigny Klaber, Melissa Orozco, Si Ning Chan, Jennifer J. Fowler, Shannon L. Gearhart, Molly B. Nicholson, Rachel Herlihy

**Affiliations:** ^1^Colorado Department of Public Health and Environment; ^2^Denver Department of Public Health & Environment, Denver, Colorado; ^3^El Paso County Public Health, Colorado Springs, Colorado; ^4^Arapahoe County Public Health, Centennial, Colorado; ^5^Boulder County Public Health, Boulder, Colorado; ^6^Travel Risk Assessment and Mitigation Branch, Division of Global Migration Health, National Center for Emerging and Zoonotic Infectious Diseases, CDC.

**Summary**

**What is already known about this topic?**

Measles is a highly contagious vaccine-preventable disease. Two measles, mumps, and rubella (MMR) vaccine doses are 97% effective in preventing measles.

**What is added by this report?**

During May 25–June 7, 2025, nine secondary measles cases and one tertiary case occurred, including four patients who were hospitalized, among Colorado residents exposed during an international flight or in an airport to an air traveler with infectious measles who had acquired the disease in the United States. Seven additional cases were reported by other jurisdictions. In two vaccinated patients, the virus was detected only in urine and not in nasopharyngeal specimens.

**What are the implications for public health practice?**

All eligible persons should receive 2 MMR vaccine doses. Travelers should ensure that their MMR vaccinations are current. Collecting urine specimens might enhance case finding, especially in vaccinated persons.

## Abstract

Measles is a highly contagious vaccine-preventable viral disease. Successful vaccination programs resulted in limited measles transmission in the United States in 2000, but U.S. cases have been increasing since early 2025. On May 20, 2025, CDC notified the Colorado Department of Public Health and Environment of a measles case in an unvaccinated, non-Colorado resident who had arrived in Denver, Colorado, on an international flight and traveled through the Denver International Airport while infectious. The patient acquired measles in the United States before travelling internationally. Nine secondary measles cases and one tertiary case associated with this traveler were confirmed among Colorado residents; seven additional cases were reported by other jurisdictions. Four of the nine secondary Colorado cases occurred among persons who had received 2 doses of measles, mumps, and rubella vaccine before exposure. Two of these vaccinated persons received negative measles reverse transcription–polymerase chain reaction (RT-PCR) test results from nasopharyngeal swab specimens and positive results from urine specimens. A third patient, with unknown measles vaccination status, received a positive urine RT-PCR test result 24 days after rash onset. Three unvaccinated patients and one with unknown vaccination status were hospitalized, and all recovered. All patients reported having a rash, but vaccinated patients reported fewer and milder symptoms overall. This outbreak highlights the importance of staying up to date with recommended vaccinations, especially before traveling. Routinely collecting urine specimens for measles testing could improve identification of cases and increase detection sensitivity, especially among previously vaccinated persons.

## Investigation and Results

Measles is a highly contagious vaccine-preventable viral disease, characterized by fever, rash, cough, coryza, and conjunctivitis. During 2025, the number of U.S. measles cases increased after 25 years of limited domestic transmission (*1*). In 2025, a total of 2,255 measles cases (including three deaths) had been reported in the United States and 11% of patients were hospitalized, the highest number of measles cases since 1992, when 2,126 cases occurred (MeaslesCasesandOutbreaks|CDC). Complicationsoccur in approximately 10% of patients with measles, including ear infections and diarrhea; serious complications including pneumonia (5%), encephalitis (0.1%), and death (0.1%–0.3%) also occur. Receipt of measles, mumps, and rubella (MMR) vaccine is the most effective way to prevent measles.* One dose of MMR vaccine is 93% protective against measles, and 2 doses are 97% protective (*2*). Although infections do occasionally occur in vaccinated persons, these cases often result in a modified illness with fewer or milder symptoms and lower rates of hospitalization (*3**,**4*). This report describes measles cases in Colorado residents that were part of an outbreak resulting from exposure to a patient who was infectious during air travel. This activity was reviewed by the Colorado Department of Public Health and Environment (CDPHE) and CDC, deemed not research, and was conducted consistent with applicable federal law, CDPHE policy,^†^and CDC policy.^§^

### Index Case Identification

On May 20, 2025, CDC notified CDPHE of a person who traveled through the Denver International Airport while infectious with measles. The person was not a Colorado resident and had not been vaccinated against measles. The traveler was exposed to measles in another state during an ongoing outbreak before traveling internationally and was reportedly symptomatic with fever and cough during the return trip. The traveler arrived in Denver on May 13 after an 11-hour international flight, stayed overnight at a Denver hotel, then returned to the airport the next day (May 14) and boarded a domestic flight to another state. During May 27–June 16, nine secondary cases and one tertiary case associated with this traveler were identified among Colorado residents. Six additional secondary cases and one tertiary case were reported by five other states. This report describes the Colorado cases and the Colorado public health response to the outbreak.

### Identification of Secondary Cases from Exposure on the International Flight

Colorado health care providers are required to immediatelyreportcasesofsuspectedmeasles to local public health authorities or CDPHE by telephone irrespective of whether laboratory results are available. On May 27, 2025, 2 weeks after the index patient arrived in Denver on the international flight, two measles cases in Colorado residents were reported to CDPHE by separate local health care providers. During the initial investigation, it was learned that these two patients had recently traveled on the same international flight as the index patient. The first confirmed case (rash onset May 25, 12 days after the flight) occurred in an unvaccinated young child (patient A) who was seated on the parent’s lap, more than two rows away from the index patient (Table) (Figure). The second case (rash onset May 26) occurred in an adult with documentation of receipt of 2 MMR vaccine doses (patient B), who was seated within two rows of the index patient. Measles in patient A was confirmed by reverse transcription–polymerase chain reaction (RT-PCR) testing of nasopharyngeal (NP) swabs and urine, and in patient B, by RT-PCR testing of NP swabs only; urine test results were negative. Urine collection for measles testing is recommended in Colorado to improve sensitivity of testing. Both patients had measles immunoglobulin M (IgM) detected by immunofluorescence assay (IFA) and enzyme-linked immunosorbent assay (ELISA).^¶^

**TABLE:** Age, vaccination status, clinical characteristics, and laboratory test results for patients with measles cases associated with an infectious air traveler — Denver, Colorado, May–June 2025

Patient*	Age group, yrs	Measles vaccination^†^	Exposure source	Rash onset date, time from exposure to onset	Signs and symptoms^§^	Hospitalized	Rash onset to specimen collection, days	Laboratory results		
								NP swab PCR^¶^	Urine PCR^¶^	IgM
A	1–9	No	Flight**	May 25, 12 days	Fever, rash, cough, coryza, conjunctivitis, and Koplik spots	Yes	2	Positive	Positive	Positive
B	30–39	Yes	Flight**	May 26, 13 days	Fever, rash, cough, coryza, and conjunctivitis	No	3	Positive	Negative	Positive
C	40–49	Yes	Flight**	May 31, 18 days	Fever, rash, and conjunctivitis	No	−1^††^	Negative	Positive	NC
D	30–39	No	Denver airport	May 27, 13 days	Fever, rash, cough, coryza, conjunctivitis, and Koplik spots	Yes	4	NC	Positive	Positive
E	30–39	No	Denver airport	May 30, 16 days	Fever, rash, cough, coryza, and conjunctivitis	No	1	Positive	NC	NC
F	20–29	Yes	Denver airport	May 30, 16 days	Fever, rash, and congestion	No	1	Positive	NC	Positive
G	20–29	Yes	Flight**	May 30, 17 days	Fever, rash, and cough	No	3	Negative	Positive	NC
H^§§^	20–29	No	Denver airport	Jun 7, 25 days	Fever, rash, cough, coryza, conjunctivitis, and Koplik spots	Yes	0	Positive	Positive	NC
I	≥50	Unknown	Denver airport	May 25, 11 days	Fever, rash, cough, coryza, and conjunctivitis	Yes	24	NC	Positive	Positive
J^¶¶^	20–29	Yes	Patient I	Jun 7, 9–17 days	Fever, rash, coryza, and conjunctivitis	No	0	Positive	NC	NC

**Abbreviations**: IgM = immunoglobulin M; NC = not collected; NP = nasopharyngeal; PCR = polymerase chain reaction.

* Patients are listed in the order in which they were identified by public health authorities.

^†^ Vaccinated patients identified in the outbreak had received 2 doses of measles-containing vaccine >2 weeks before exposure.

^§^ This includes measles signs and symptoms documented in medical records, specifically fever, rash, cough, coryza, conjunctivitis, and Koplik spots. Other signs, symptoms, or complications are not included.

^¶^ NP and urine specimens were collected on the same day.

** Same international flight as index patient.

^††^ Patient C was contacted by the local public health agency as part of the routine air contact investigation. The patient reported not feeling well and did not have a rash at the time specimens were collected for testing. A rash was identified the day after specimen collection and described as acne-like.

^§§^ Although no other exposures ≤21 days of rash onset were identified for patient H, they cannot be ruled out.

^¶¶^ Patient J was identified as a household contact of patient I and had a tertiary case of measles. Because of frequent exposures during patient I’s infectious period, days from exposure to rash onset is presented as a range.

**FIGURE:**
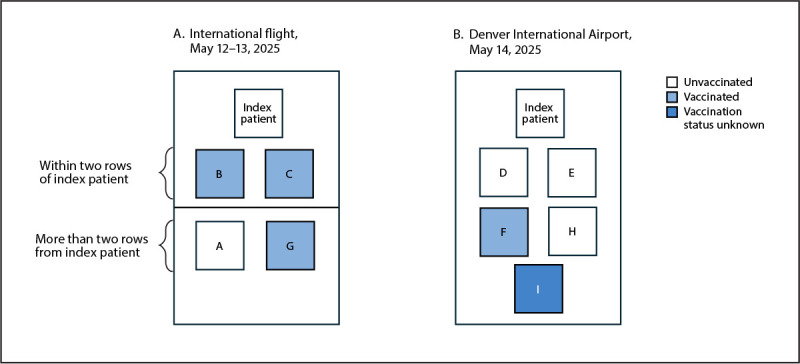
Vaccination status* of patients with secondary measles^†^ who were exposed to the index patient during an international flight (A) or in an airport^§^ (B) — Denver, Colorado, May–June 2025 * Vaccinated persons had documentation of receipt of 2 doses of measles, mumps, and rubella vaccine. ^†^ Positions of persons with secondary cases do not indicate their exact location relative to the index patient or to one another. A 10th (tertiary) measles case that occurred in a household contact of patient I is not shown. ^§^ Exposure in the main terminal or a specific concourse.

On May 28, after CDPHE notified CDC of the cases (which CDC considered to be part of an outbreak in the state where the index patient was initially exposed), CDC provided CDPHE with the list of Colorado residents identified as airplane contacts from the international flight, which included patients A and B. CDC routinely includes children seated on a parent’s lap anywhere on an airplane, persons seated within two rows of a person with measles on an airplane, and crew members serving the infected person in airplane exposure notifications for flights with a capacity of more than 50 passengers. For flights with a capacity of 50 or fewer passengers, all passengers and crew members on board are included in notifications. CDPHE distributed the air contact list to the respective local public health agencies based on contacts’ addresses so that these travelers could be assessed for measles immunity and presence of symptoms. During these interviews, a third (vaccinated) person (patient C), who was also seated within two rows of the index patient, reported developing respiratory symptoms without rash on May 29; this patient then developed a rash on May 31. NP swab and urine specimens were collected on May 29, 2 days before rash onset. RT-PCR testing of urine confirmed measles, and the NP swab was negative.

Because three secondary cases were identified on this flight, CDPHE requested an expanded air contact list that included passengers seated more than two rows away from the index patient. This expanded list was received on May 30; based on this list, an additional measles case was identified in a vaccinated person (patient G) who was seated five rows from the index patient. No other measles cases were identified among Colorado residents from the international flight.

On May 22, CDC provided CDPHE with a list of airplane contacts who were Colorado residents from the domestic flight; no measles cases were identified among Colorado residents from that flight. CDPHE distributed a healthalertnetwork(HAN) message on May 30 to alert Colorado health care providers of confirmed measles cases identified among Colorado residents who were exposed to the out-of-state index traveler during an international flight. This HAN referenced a May 23 HAN that provided notification of the index traveler’s movement through the airport and Denver hotel stay on May 13 and 14.

### Identification of Secondary Cases from Exposure in the Denver Airport and a Tertiary Case

On May 31, local providers reported three additional measles cases among adults aged 20–39 years (patients D, E, and F) with rash onset on May 27 (patient D) and May 30 (patients E and F). All three patients reported being at the Denver airport on May 14, the same day the index patient boarded a domestic flight at the airport; among these patients, one (patient F) was vaccinated. On June 7, a provider reported another measles case (patient H) in an unvaccinated person employed by an airport business, who worked a shift on May 13, the day the index patient arrived in Denver. Patient H had first symptom onset on June 2 and rash onset on June 7; RT-PCR results from both urine specimen and NP swab were positive. Although the rash onset occurred 4 days outside the maximum expected incubation period of 21 days, first symptom onset was on day 20 of the incubation period; no other potential exposures between May 13 and the onset of symptoms were identified for this patient.

On June 16, an adult aged ≥50 years with unknown vaccination status (patient I) contacted public health officials and reported a recent hospitalization for acute hepatitis during May 27–29 and concern about possibly having measles after learning that a household contact had measles. Patient I reported rash onset on May 25. On further investigation, the patient was confirmed to have been at the Denver airport on May 14 in the same terminal as the infectious traveler but not on any of the same flights. The patient had had signs and symptoms consistent with measles before and during hospitalization; however, measles was not suspected, and testing was not performed during the hospitalization. Measles was confirmed on June 18 (24 days after rash onset) by PCR testing of urine after the patient had recovered and been discharged from the hospital.

The investigation of patient I’s case revealed that the measles case reported to CDPHE on June 12 in a patient with rash onset on June 7 and a positive NP RT-PCR test result (patient J) had occurred in a household contact of patient I. Patient J’s illness was initially believed to be unrelated to the ongoing outbreak because the rash began on June 7, and although the patient reported international travel (May 15–May 21), no Denver airport transit days coincided with the days the index patient was in the airport (May 13 and 14). Patient J’s case was later classified as a tertiary case, having been infected by patient I.

Four of the nine secondary cases were passengers on the same international flight as the index patient, including three who were vaccinated (Table). An additional five secondary cases occurred among persons who were exposed to the index patient in the Denver International Airport main terminal and a specific concourse, but not on a flight; one of these five persons was vaccinated, three were unvaccinated, and the vaccination status of one was not known. One person was an employee at a business in the main terminal who was likely exposed on the date of arrival of the international flight. A CDC investigation of the domestic terminal determined that the other four cases likely resulted from exposures the following day in a smaller, more congested concourse where the index patient boarded a domestic flight.

### Laboratory Testing

All measles cases were laboratory confirmed by RT-PCR testing of urine or NP specimens at the Colorado State Public Health Laboratory or at commercial laboratories. Urine RT-PCR results were positive in six patients, including two vaccinated patients who received negative NP test results (patients C and G) (Table). In patient I, the hospitalized patient whose vaccination status was unknown, measles virus was detected in urine by RT-PCR testing 24 days after rash onset; no NP specimen was collected. Because the investigation of this case occurred outside the recommended time frame for measles specimen collection (i.e., >14 days after rash onset), measles IgM testing was recommended; an ELISA test detected IgM, indicating recent infection. Among six genotyped specimens, all were D8, the most commonly circulating genotype identified in recent 2025 U.S. outbreaks (*5*).

### Characteristics of Patients with Secondary Measles Cases

Among the nine patients with secondary cases in Colorado, four had received 2 MMR vaccine doses ≥2 weeks before exposure, four were unvaccinated, and the vaccination status of one was unknown (Table). The median patient age was 29 years (range = 1–55 years). All nine patients experienced rashes of varying severity. Two vaccinated patients reported mild, atypical rashes, which they compared with acne or insect bites. Vaccinated patients generally reported fewer and milder symptoms than did unvaccinated patients.

Three unvaccinated patients (including the young child from the international flight [patient A] and the two Denver airport contacts [patients D and H]), and one patient with unknown vaccination status (patient I) were hospitalized for 2–5 days with severe signs, symptoms, and complications, including high fever, dehydration, diarrhea, anorexia, and acute viral hepatitis; no deaths occurred. The median age of hospitalized patients was 30 years (range = 1–55 years).

## Public Health Response

For every confirmed measles case in Colorado, CDPHE conducts a full contact investigation, including identification and notification of all potentially exposed persons to assess the need for postexposure prophylaxis (PEP) and to monitor susceptible persons during their incubation period** (*2*). Approximately 1,400 contacts of the patients with primary, secondary, and tertiary measles cases were identified in Colorado. Public health officials attempted to reach all 1,400 contacts by telephone, text message, or both.

A list of 92 airplane contacts was provided to CDPHE from CDC for both flights. Local public health agencies made at least three telephone attempts to reach persons potentially exposed on the flight and to collect information on measles immunity and any measles signs or symptoms. Measles immunity was assessed based on documentation of receipt of ≥1 age-appropriate MMR vaccine dose, laboratory evidence of immunity, laboratory confirmation of disease, or birth before 1957. Household contacts and health care personnel who had close, prolonged contact with a patient were considered high-risk close contacts. Among persons who did not have close, prolonged contact with a patient with measles and who did not have presumptive evidence of immunity (*2*), public health officials considered 1) verbal report of having received MMR vaccination, 2) service in the U.S. armed forces, or 3) entry into the United States with an immigrant visa or a green card as adequate evidence of immunity to avoid quarantine (ImmigrantandRefugeeHealth|CDC). Persons who could not be reached by telephone were sent email messages and postal messages. If immunity to measles could not be verified, public health officials monitored contacts through telephone calls, text messages, or online surveys for 21 days from the time of exposure (i.e., one measles incubation period) for development of any measles signs or symptoms. If immunity was verified, contacts were given information about signs and symptoms of measles and were advised to self-monitor for 21 days and to contact public health authorities or their health care provider (before seeking care) if they experienced any measles-compatible signs or symptoms. Among the 92 airplane contacts, 74 (80%) were assessed; 18 could not be contacted after at least three attempts within a week and were considered lost to follow-up. All airplane contacts were identified after the recommended time for administration of PEP had passed; therefore, no airplane contacts were recommended to receive PEP. Sixty-one contacts of the patients with secondary and tertiary cases were identified as being eligible to receive PEP. Among these contacts, 12 (20%) were recommended to receive immune globulin, 10 of whom received it (two declined). MMR vaccine was recommended as PEP for 49 (80%) contacts, 41 (84%) of whom received it. Six (12%) contacts for whom MMR vaccine was recommended declined it, and for two (4%), information about whether MMR vaccine was received is not known. Apart from the 10 previously identified secondary and tertiary measles cases, no additional cases were detected.

HAN messages, press releases, and updates to CDPHE’s webpage were used to notify the public of all known locations where exposure to any of the 10 Colorado measles patients with secondary and tertiary measles during their infectious period might have occurred. Public health agencies worked with the facilities where three of the four hospitalized patients had been admitted to notify health care personnel and other patients who might have been exposed about the potential need for PEP.

To identify contacts of patient I, who had been hospitalized during the infectious period and whose measles infection case was not recognized until the patient notified public health authorities after hospital discharge, a case-finding approach was implemented to survey health care providers and patients who were exposed during the patient’s hospitalization and to ascertain whether anyone experienced measles symptoms. The only tertiary case identified was patient J, a household contact of patient I, whose case had been reported before patient I’s case was identified. Rash onset for patient I occurred on June 7, and the end of the outbreak in Colorado was considered to be July 23, 2025, two incubation periods (or 42 days) from the last infectious date of this tertiary case.

## Discussion

Although secondary cases of measles among flight contacts are rare (*6*), nine secondary cases were identified among Colorado residents exposed to a person with infectious domestically acquired measles on an international flight or in the airport. No Colorado cases were identified on the domestic flight. The duration of the international flight (11 hours) might have contributed to measles transmission, including to vaccinated persons; in addition, the index patient was reported to have had a fever and to have been coughing during travel. Identification of air travel exposure allowed public health officials to assess airplane contacts and provide disease control recommendations as well as to link airport exposures to this index patient and contribute to the understanding of this outbreak.

During this investigation, testing of urine specimens enhanced case finding, especially among vaccinated persons, who might have experienced milder symptoms and potentially received a negative measles IgM test result (*4*,*7*). NP and throat swabs are often preferred over urine specimens for measles testing; collection and testing of urine specimens is not routinely recommended if NP and throat swabs have been collected (*8*). In this investigation, two patients who received negative RT-PCR test results from NP swabs received positive urine test results, suggesting that urine specimens might have the potential to increase sensitivity for and the likelihood of detecting measles virus, especially among vaccinated persons (*9*).

### Limitations

The findings in this report are subject to at least two limitations. First, the findings included in this report are limited to cases among Colorado residents and therefore do not reflect the magnitude and public health impact of this outbreak across other jurisdictions. The seven additional cases among residents of other jurisdictions and their contacts were followed up by their local jurisdictions. Second, reaching all potentially exposed persons in this outbreak was limited by public health resources; CDPHE and local public health agencies prioritized contact investigations based on disease control strategies such as prioritizing contacts who were eligible for PEP. However, because measles is a reportable disease and information about the cases was shared widely with public health and clinical partners, in the absence of additional reported cases, it is unlikely that transmission continued after the second generation aside from the one tertiary case.

### Implications for Public Health Practice

Measles is a highly infectious disease (secondaryattackrate≥90%) but is vaccine preventable. Host infectiousness, duration of exposure, and crowded settings probably contributed to measles transmission in this outbreak (*7*,*10*), highlighting the risk for measles transmission from infectious travelers in both planes and airports and the value of ensuring that all eligible persons, particularly travelers, receive 2 doses of measles-containing vaccine to protect against measles. Collection of urine for measles RT-PCR testing might increase sensitivity of case finding, particularly in vaccinated persons.

## Footnotes

* Persons with documentation of receipt of ≥1 dose of MMR vaccine on or after their first birthday and ≥2 weeks before the exposure were considered vaccinated.

† Colorado Revised Statutes Sect. 25–1-122 and Colorado Code of Regulations at 6 CCR 1009–1.

§ 45 C.F.R. part 46.102(l)(2), 21 C.F.R. part 56; 42 U.S.C. Sect. 241(d); 5 U.S.C. Sect. 552a; 44 U.S.C. Sect. 3501 et seq.

¶ IFA testing was performed at MayoLab. ELISA testing was performed at AssociatedRegionalandUniversityPathologists,Inc.(ARUPLaboratories).

** Postexposure prophylaxis for measles includes the MMR vaccination ≤72 hours of initial exposure to measles, or, for some persons, immune globulin can be offered ≤6 days of initial exposure.
